# Sensitivity to musical emotion is influenced by tonal structure in congenital amusia

**DOI:** 10.1038/s41598-017-08005-x

**Published:** 2017-08-08

**Authors:** Cunmei Jiang, Fang Liu, Patrick C. M. Wong

**Affiliations:** 1 Music College, Shanghai Normal University, Shanghai, China; 2 Institute of Psychology, Shanghai Normal University, Shanghai, China; 3 School of Psychology and Clinical Language Sciences, University of Reading, Reading, UK; 4 Department of Linguistics and Modern Languages and Brain and Mind Institute, The Chinese University of Hong Kong, Hong Kong, China; 5 The Chinese University of Hong Kong – Utrecth University Joint Center for Language, Mind and Brain, Hong Kong, China

## Abstract

Emotional communication in music depends on multiple attributes including psychoacoustic features and tonal system information, the latter of which is unique to music. The present study investigated whether congenital amusia, a lifelong disorder of musical processing, impacts sensitivity to musical emotion elicited by timbre and tonal system information. Twenty-six amusics and 26 matched controls made tension judgments on Western (familiar) and Indian (unfamiliar) melodies played on piano and sitar. Like controls, amusics used timbre cues to judge musical tension in Western and Indian melodies. While controls assigned significantly lower tension ratings to Western melodies compared to Indian melodies, thus showing a tonal familiarity effect on tension ratings, amusics provided comparable tension ratings for Western and Indian melodies on both timbres. Furthermore, amusics rated Western melodies as more tense compared to controls, as they relied less on tonality cues than controls in rating tension for Western melodies. The implications of these findings in terms of emotional responses to music are discussed.

## Introduction

Congenital amusia (hereafter amusia) is a lifelong disorder of music processing, which cannot be attributed to prior brain lesion, hearing loss, or any cognitive or socioaffective disturbance or lack of exposure to music^1^. Individuals with amusia have been characterized by deficits in fine-grained pitch discrimination^2–4^, and melodic contour and pitch direction discrimination^2, 5, 6^. Despite showing intact implicit structural processing in music^7^, amusics demonstrate deficits in processing musical syntax and tonality in an explicit manner^8^. The dissociation between implicit and explicit processing of musical structure has been confirmed by other studies^9–11^. Such a dissociation has also been reported for pitch processing, in which intact early pre-attentive processing of fine-grained pitch fails to reach the level of conscious processing in amusia^12, 13^.

Emotional communication is a pivotal function for music. Despite their pitch perceptual deficits, amusic individuals do not always show impairments in music appreciation^14^. Indeed, it has been suggested that amusics are sensitive to acoustic differences between consonance and dissonance^15, 16^, owing to their sensitivity to roughness^16^. Two recent studies^17, 18^ which explored the processing of musical emotion in amusia converged on similar conclusions. Marin *et al*.^17^ demonstrated that amusics’ affective responses to isolated dyads and triads were affected by timbre. For instance, amusics showed some sensitivity to the association between major mode and happiness in the complex-tone, but not in the sine-tone condition. This difference may result from amusics’ deficits in the perception of harmonicity, since the two timbres (complex-tone and sine-tone) differed in harmonic spectra. Similarly, Gosselin *et al*.^18^ reported that amusics were able to recognize musical emotions as well as controls in the real music context, because emotional classifications of those stimuli could be distinguished on the basis of acoustic characteristics. For example, the happy excerpts were in a major mode and were played at a relatively fast tempo, while the sad excerpts were in a minor mode and were played at a relatively slow tempo. Even when the mode was inverted, amusics were still able to recognize emotions based on temporal cues that remained available in the mode-change version^18^.

Indeed, emotional connotations of music can be conveyed through psychoacoustic cues such as tempo, melodic contour, harmonic complexity, melodic complexity, rhythmic complexity, consonance/dissonance, and timbre, and these attributes of sound can be perceived without implicit or explicit knowledge of tonal system information^19^. Using these psychoacoustic cues, human and nonhuman species have shown similarities in encoding and decoding emotional signals in acoustic communication^20–22^. Even for unfamiliar cross-cultural music, listeners are able to recognize musical emotion on the basis of these cues^19, 23–25^. Therefore, it is not surprising that amusics showed normal sensitivity to musical emotion since their emotional recognition could be facilitated by temporal characteristics and timbre in Gosselin *et al*.^18^. However, owing to the deficits in the perception of harmonicity, amusics were impaired in emotional evaluations based on harmonicity (but not on roughness), as revealed by Marin *et al*.^17^.

The influence of psychoacoustic cues on emotional communication is not, however, unique to music. For example, changes in tempo, loudness, rhythm, and pitch also impact emotional connotations in speech prosody^26^ and in environmental sounds^27^, which is related to sensory perception. It is the tonal system that makes music unique in emotional communication. Although tonal system information may assist in accurate recognition of emotional intentions, e.g., through the link between a major key and the feeling of happiness, it affects musical emotion mainly through tension-resolution patterns. Specifically, melodic and harmonic progression, and global or local syntactic structure governed by functionally tonal regularities may fulfill or violate musical expectations^28, 29^, and these violated or fulfilled expectations create musical tension or resolution^30, 31^.

Tension, a dimension in the three-dimensional model of affect described by Schimmack and Grob^32^, is also an emotional category. Apart from the influences of acoustic cues such as timbre, tempo, and sensory dissonance^33, 34^, the general tension-resolution pattern of music is primarily influenced by tonal structure^35–39^. A cross-cultural study of music has also found the role of tonal system on emotion perception, with monocultural listeners but not bicultural listeners showing an in-culture advantage for their own music in tension judgment^40^. Based on tonal structure rather than acoustic cues, such tension judgments correspond to a cognitive level beyond acoustics since they express a state of instability^37^. It can arise through the internal knowledge of tonal system, acquired from prior musical exposure and perceptual learning^40^. Given the dissociation between implicit and explicit tonal structure processing in amusia^7, 8, 10, 11^, tension perception may provide us with an ideal opportunity to examine amusics’ sensitivity to musical emotions, particularly to those elicited by tonal structure information.

Thus, the aim of the present study was to investigate musical tension processing in individuals with congenital amusia. Both timbre and tonal system were manipulated, which allowed us to compare the capacity of individuals with amusia to decode emotional meaning through psychoacoustic and tonal cues, respectively. Melodies composed in Western and Indian music systems were used. Given amusics’ impairment in harmonicity perception^16, 17, 41^, we included both piano and sitar timbres in the present study. This design ensured that the melodies played on the two instruments differed not only in harmonicity, but also in other timbre information such as roughness. Fifty-two Mandarin speakers with and without amusia, diagnosed by the Montreal Battery of Evaluation of Amusia (MBEA)^42^, listened to the melodies and rated the tension evoked by these melodies using a Continuous Response Digital Interface dial with the scale of 0 to 255^43^.

Since Western tonal music was introduced to China in the early twentieth century, listening to tonal music (including Chinese music written in Western tonal system) has become an essential experience for Mandarin listeners, either in the curricular or extracurricular environment^44^. Thus, participants in our study were typically familiar with Western music and piano timbre, the latter of which is widely used in Western music works. Furthermore, none of our participants reported that they were familiar with Indian music or instruments, which made it impossible for them to perceive the culture-specific feature of sitar as an Indian instrument. Consequently, the piano and sitar timbres in the present study would be interpreted as an acoustic difference. Indeed, unlike piano, sitar contains plucked strings and a curved bridge, and the interaction between the string and the bridge generates high frequency components, which creates a buzzing tone^45^ with a feeling of dissonance^46^. Given that timbre affects the perception of emotion in music, independently of cognitive factors^47^, we expected that both the amusic and control groups would use timbre cues to judge tension in Western and Indian music. Given that emotional connotations evoked by a tonal system can be recognized only by listeners familiar with the musical culture^19, 34^, we expected that controls would rate Western and Indian music differently in tension. However, amusics would not exhibit this expected familiarity effect for Western music because they have reduced sensitivity to tonality of Western music^8^.

## Results

**Figure 1:**
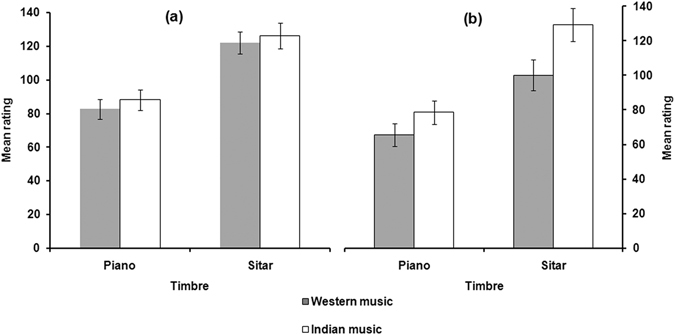
Tension judgment for the Western-piano, Western-sitar, Indian-piano, and Indian-sitar conditions by amusics (**a**) and controls (**b**). Error bars indicate standard errors.

Figure 1 presents tension ratings of the amusic and control groups. The y-axis shows a continuous but arbitrary unit of tension corresponding to the scale of the Continuous Response Digital Interface dial that the participants used to rate tension. As can be seen, unlike controls, amusics did not exhibit obvious different ratings for Western versus Indian melodies.

Since controls’ ratings in the Western-piano condition showed a non-normal distribution (Shapiro-Wilk *W* = 0.86, *p* = 0.002), we transformed our data into logarithmic form for the subsequent analysis of variance (ANOVA). A three-way mixed-factor ANOVA taking group (amusics versus controls) as the between-subjects factor, and music culture (Western versus Indian) and timbre (piano versus sitar) as the within-subjects factors revealed significant main effects of music culture, F (1, 50) = 9.87, *p* = 0.003, η_p_^2^ = 0.17, and timbre, F (1, 50) = 81.83, *p* < 0.001, η_p_^2^ = 0.62. There was a significant two-way interaction between music culture and group, F (1, 50) = 5.41, *p* = 0.02, η_p_^2^ = 0.10, reflecting that controls assigned different tension ratings to Western and Indian melodies, F (1, 50) = 14.95, *p* < 0.001, η_p_^2^ = 0.23, while amusics showed comparable tension ratings for Western and Indian melodies, F (1, 50) = 0.33, *p* = 0.57, η_p_^2^ = 0.007. Planned comparisons also revealed amusics rated Western melodies as more tense than controls, F (1, 50) = 7.04, *p* = 0.01, η_p_^2^ = 0.12, whereas the two groups showed comparable ratings on Indian melodies, F (1, 50) = 0.44, *p* = 0.51, η_p_^2^ = 0.01. Furthermore, a main effect of group, F (1, 50) = 3.14, *p* = 0.08, η_p_^2^ = 0.06, and a three-way interaction between music culture, timbre, and group, F (1, 50) = 3.57, *p* = 0.07, η_p_^2^ = 0.03, reached a marginally significant level. Other interactions were not significant (*p*s > 0.27).

There were 5 participants (3 amusics and 2 controls) who provided the highest ratings in one of the four conditions, which were two standard deviations above the mean rating of their own groups. However, when these participants were removed from the analysis, the results showed the same pattern as above. Moreover, to avoid the effect of individual differences on mean ratings of the participants, the differences in rating for tension on each trial were individually normalized to z-scores and subjected to the same analyses. The patterns of results still remained the same as above.

Another factor that might have affected tension ratings in our participants was the different endings (tonic, in-key, and out-of-key tones) we included in our melodies, which were to elicit overall greater tension response. In order to examine this effect, we conducted a four-way mixed-factor ANOVA with ending as an additional factor. The main results involving the other factors showed the same pattern as above. In addition, we found a significant main effect of ending, F (1.83, 91.47) = 6.13, *p* = 0.003, η_p_^2^ = 0.11, which was due to a significant linear trend, F(1, 25) = 8.80, p = 0.05, η_p_^2^ = 0.15, and a marginally significant two-way interaction between music culture and ending, F (2, 100) = 2.99, *p* = 0.055, η_p_^2^ = 0.06. This result indicates that both amusic and control participants rated tension differently depending on the three different ending tones.

Spearman correlations were calculated between participants’ tension ratings in the four experimental conditions and their MBEA global and three pitch-based subtests scores for each group. Tension ratings of Western and Indian music on piano or sitar were not significantly correlated with any subtest of the MBEA, global or the three pitch-based subtests scores for the control group (*p*s > 0.15). For the amusic group, however, there was a significant correlation between the scores of the scale subtest and the ratings of Indian music on sitar (*r*_s_ = −0.45, df = 24, *p* = 0.02). Moreover, there were 9 amusics and 8 controls who also participated in our previous study on musical syntactic processing (Jiang *et al*.^8^). We calculated the correlation between the performances on the two studies for amusics and controls, respectively. For amusics, musical syntactic performances were significantly correlated with tension ratings only in the Western-piano condition, *r*(7) = 0.74, *p* = 0.03, but not in the Western-sitar condition, *r*(7) = 0.52, *p* = 0.15. For controls, their syntactic performances were not significantly correlated with tension ratings in either the Western-piano, *r*(6) = 0.19, *p* = 0.66, or the Western-sitar condition, *r*(6) = 0.25, *p* = 0.55. These findings suggest that amusics might use different strategies from controls in rating tension.

In order to examine whether amusics used different strategies from controls in rating tension, 14 parameters related to timbre and 4 parameters related to tonality of each melody were extracted using MIR toolbox 1.6.1^48^. Linear regressions were conducted to investigate which timbre and tonality cues predicted tension ratings of the two groups. There was no multicollinearity in the models, since none of the correlations between the predictors was significant, whether for timbre-related or for tonality-related parameters (all *p*s > 0.05). Table 1 presents the timbre-related and tonality-related parameters that contributed significantly to tension ratings in Western and Indian melodies. As can be seen, while the ratings of controls were predicted by the tonality-related parameters such as harmonic chord detection function (HCDF) in both the Western-piano and Western-sitar conditions, amusics’ ratings were predicted by HCDF only in the Western-piano condition. Moreover, *R* ^2^ values in amusics were smaller than those in controls for the Western-piano condition. Following Cohen^49^, *R* ^2^ can be translated into f^2^, an effect size index for regression and correlation analysis. The effect size for controls was large (f^2^ = 0.67 for Western-piano), while the effect size for amusics was small (f^2^ = 0.14), since f^2^ = 0.02 reflects a small effect size, and 0.15 and 0.35 reflect a medium and a large effect size, respectively. Even for the timbre cues, tension ratings by amusics and controls relied on different parameters, e.g., zero-cross rate versus spectral flux in the Western-sitar condition, as shown in Table 1. Although the MIR toolbox applies to Western tonal music, controls’ ratings in the Indian-sitar condition were predicted by HCDF, whereas amusics’ ratings for the Indian melodies were not predicted by any tonality cues. The former finding suggests that long-term schematic knowledge of the Western music system (familiar to Mandarin listeners) might affect tension perception of Indian music (unfamiliar to Mandarin listeners) since there was an overlap of the used tones between Western and Indian tonal scales. Regarding timbre cues, similar to the ratings for Western melodies, the two groups used different timbre cues for tension rating, e.g., attack time versus spectral flux in the Indian-sitar condition.

**Table 1:** The timbre-related and tonality-related cues that contributed significantly to tension ratings in the Western and Indian music conditions.

	Amusic Group						Control Group					
	Predictor	*R* ^2^	*F*	*p*	*β*	*p*	Predictor	*R* ^2^	*F*	*p*	*β*	*p*
Tonality in WP	HCDF	0.12 (0.14)	6.29	<0.05	0.38	<0.05	HCDF	0.40 (0.67)	27.35	<0.001	0.65	<0.001
Timbre in WP	Spectral flux	0.13 (0.15)	5.79	<0.05	0.36	<0.05	Spectral flux	0.46 (0.85)	34.424	<0.001	0.68	<0.001
Tonality in WS	None						HCDF	0.31 (0.45)	17.28	<0.001	0.56	<0.001
Timbre in WS	Zero-cross rate & irregularity	0.26 (0.35)	7.98	0.001			Spectral flux & Roughness	0.37 (0.59)	12.55	<0.001		
	Zero-cross rate				0.42	<0.01	Spectral flux				0.57	<0.001
	Irregularity				0.26	0.07	Roughness				0.18	0.17
Tonality in IP	None						MFCC	0.13 (0.35)	5.60	<0.05	0.36	<0.05
Timbre in IP	None						None					
Tonality in IS	None						HCDF	0.14 (0.16)	7.52	<0.01	0.41	<0.01
Timbre in IS	Attack time	0.14 (0.16)	6.34	0.016	−0.38	0.016	Spectral flux	0.26 (0.35)	13.26	0.001	0.51	0.001

Cohen’s f^2^, an effect size index for regression analysis, are shown in parentheses. WP = Western-piano, WS = Western-sitar, IP = Indian-piano, and IS = Indian-sitar.

## Discussion

Psychoacoustic attributes of sound carry emotional connotations across channels of acoustic communication^19, 26, 27^. What tonal system information conveys towards emotion, however, is unique to music. We tested a relatively large sample of Mandarin-speaking amusics and matched controls on tension ratings of Western and Indian melodies played on piano and sitar. The manipulations of timbre and tonal system were important because this allowed us to compare the capacity of individuals with amusia to decode emotional meaning through psychoacoustic and tonal cues, respectively. Both amusics and controls gave higher tension ratings for both Western and Indian melodies played on sitar than piano. However, while controls rated the Indian melodies as more tense than Western melodies regardless of timbre (piano or sitar), showing a tonal familiarity effect on tension ratings, amusics showed comparable tension ratings for Western and Indian melodies. Furthermore, amusics rated Western melodies as more tense compared to controls, which may be attributed to the fact that amusics’ ratings did not rely on tonality-related cues as markedly as controls. These findings suggest reduced tonal familiarity effect for Western music in amusia.

The novel finding of our study is that unlike controls, amusics showed comparable tension ratings for Western and Indian melodies, indicating the reduced sensitivity to musical emotion evoked by tonal system in amusia. As noted earlier, Mandarin listeners acquire the knowledge of Western tonal system owing to their daily exposure to the tonal music environment. This perceptual learning may explain why the control participants distinguished Western from Indian music in the present study. Given that amusics are as well equipped with Western tonal music knowledge as controls^7, 10^, the present data indicate that the knowledge of tonal system acquired implicitly cannot help amusics develop explicit response to musical emotion, since amusics’ tonal music knowledge cannot access the conscious level^12, 13^.

Given the potential roles that familiarity plays in emotional response to music^50, 51^, familiarity may be another possible explanation for the reduced sensitivity to musical emotion in amusia. This effect may result from long-term exposure to tonal regularities. During a listening experience, the long-term exposure to tonal regularities leads to the formation of mental representations that generate online expectancies as music unfolds. Persistent violations to tonal expectations, in turn, should tend to increase perceived tension in music. Thus, music with unfamiliar tonal structure should be judged as higher in tension than music with familiar tonal structure. This point may account for the familiarity effect seen in controls, but not observed in amusics. Furthermore, despite having similar exposure to the environment of Western tonal music, amusics rated Western melodies as more tense than controls. This finding further confirms the difference in tonal familiarity effect between the two groups, and suggests that sensitivity to perceived emotion conveyed by tonal system relies on familiarity with the tonal system.

The present study is consistent with the findings of previous studies^15–18^ suggesting that amusics were able to process musical emotion conveyed by psychoacoustic information such as tempo and instrumental timbre in music. As far as familiarity is concerned, the timbre of piano is commonly perceived for Mandarin listeners during daily listening, which is different from that of sitar. The present study demonstrated that both the amusic and control groups rated the melodies on piano (familiar) as more tense than those on sitar (unfamiliar) for both Indian and Western tonal systems. These findings suggest that sensitivity to perceived emotion conveyed by psychoacoustic information does not rely on familiarity with this information, which is unlike the response to emotion evoked by tonal system information.

The present findings are in contrast to what was reported by Gosselin *et al*.^18^, in which amusics were able to recognize musical emotions, even when mode was manipulated. This discrepancy may be attributed to the differences in methodology between the two studies. First, although Gosselin *et al*.^18^ tried to manipulate mode, the temporal cues that corresponded to emotion categories (e.g., happy and sad) still remained available. Thus, their participants were able to classify musical emotions using these temporal cues. Furthermore, even for the mode manipulation, the Western major and minor scale structures in Gosselin *et al*.^18^ may have been well learned for participants through passive exposure and perceptual learning. In contrast, we manipulated tonal system by using Western and Indian melodies. Mandarin listeners are familiar with the Western tonal system, but not with the Indian tonal system. Given that the internal knowledge of tonal system is acquired from prior musical exposure and perceptual learning^40^, it would be more revealing to examine sensitivity to musical emotion by comparing the tonal systems between familiar and unfamiliar cultures than by comparing two familiar tonal structures in the same culture. Second, although both Gosselin *et al*.^18^ and our study focused on sensitivity to musical emotion, the tasks involved were different. Unlike the tasks of emotional recognition in Gosselin *et al*.^18^, we used the Continuous Response Digital Interface dial for tension rating, with the scale ranging from 0 to 255. This design may have led to more sensitive emotional response than emotional recognition in Gosselin *et al*.^18^, in particular for emotions evoked by tonal system. Indeed, it would be difficult for listeners to make accurate emotional classification based upon tonal system information alone, without the aid of psychoacoustic cues or verbal information. This is because the tension-resolution pattern may be the main response to emotions evoked by tonal system. In this case, tension rating may be an ideal approach to investigate responses to emotions exclusively evoked by tonal system. Finally, sample size may also account for the discrepancy. There were only 13 amusic participants in Gosselin *et al*.^18^, whereas we included 26 amusics in our study. A larger sample size would reflect the population mean more reliably, thus ensuring the reliability of the data.

Consistent with Gosselin *et al*.^18^ who reported a dissociation between pitch detection and emotion, the present study demonstrated that tension ratings in controls were significantly correlated neither with the scores in the three pitch-based subtests nor with the global scores of the MBEA. This finding suggests that the global tension-resolution (or emotion) perception is independent from subtle pitch discrimination and basic musical ability. It is worth noting, however, that amusics’ tension ratings in the Indian-sitar condition were significantly correlated with the scores of the scale subtest of the MBEA. This finding does not suggest that amusics’ ratings relied on their abilities to explicitly detect out-of-key tones since none of the tonal parameters predicted the ratings in the Indian-sitar condition for amusics, as revealed by the regression analysis. This significant correlation may be attributed to the similar strategies amusics used in the two tasks. For example, sensory dissonance may be used for both the performance on the scale subtest and tension judgment in the Indian-sitar condition. Future studies are needed to examine this issue.

The present study manipulated tonal system (Western versus Indian music) to assess perceived musical emotion. The findings would thus provide evidence for cross-cultural research on musical emotion. First, the finding regarding control participants corroborates our previous study by showing that monocultural typical listeners have in-culture bias for musical tension judgment^40^. This is because emotional connotations evoked by tonal system information can be recognized only by cultural insiders or listeners familiar with the musical culture^19, 34^, and such a privileged position of familiar music would result in a cultural bias of emotion perception towards familiar music. However, the absence of in-culture bias in bimusical listeners^40^ is different from amusics’ tension performance in the present study. While amusics lack the sensitivity to their familiar music system, bimusical listeners show dual sensitivity to both musical cultures^40^, which are shaped by multiple behavioral and neural factors^52^.

Second, regarding the universality of cultural sensitivity to musical emotion, although the ability to recognize basic emotions in Western music has been considered to be universal^25^, our findings do not provide positive evidence to support this view from the perspective of amusia. Specifically, consistent with previous studies suggesting the universality of using psychoacoustic cues to recognize musical emotion for familiar or unfamiliar music^19, 23–25^, both the amusic and control groups used timbre-related cues to rate tension, regardless of Western or Indian melodies. Unlike controls, however, amusics did not distinguish Western from Indian melodies when tonal system was manipulated. Given that emotion conveyed by tonal system information is unique to music, our findings indicate that the processing of musical emotion is not universal, at least for amusic individuals.

Consistent with the findings in Bigand and Parncutt^39^, tension ratings in both amusics and controls were affected by the ending tones with different stabilities. Unlike controls, however, the hierarchy of tension ratings in amusics does not mean that they could process tonal system information, because controls, but not amusics, rated Indian melodies as more tense than Western melodies. Such a hierarchy of tension ratings in amusics may reflect the processing of tone frequency. Specifically, given the absence of familiarity effect on Western tonal system, amusics might consider Western melodies as just unfamiliar as Indian melodies. Indeed, listeners can exhibit tonal hierarchies of unfamiliar musical culture based on the distribution of tones in relatively long melodies^53^. This is because tones that were given higher ratings (more stable) in the probe-tone task also sounded more frequent in music^54^. From this perspective, a relatively long melody may be necessary for the processing of tone frequency. This point can also account for the discrepancy in performances by amusics in the present study and in Jiang *et al*.^8^, the latter of which suggests reduced sensitivity to the hierarchy of stability of tones in amusia. While the present study used relatively long melodies ranging from 10 to 15 s, the key-defining context contained only a scale or a cadence with durations ranging from 800 ms to 3.5 s in Jiang *et al*. In such short musical contexts, it is unlikely for amusics to establish a hierarchy of stability of tones based on the distribution of tones. Similarly, Jiang *et al*. used 5-tone melodies with duration of 3 s for music-syntactic tasks. In this case, it is also impossible for amusics to make a syntactic judgment based upon the tone frequency, thus resulting in their reduced sensitivity to the distinction between regular and irregular endings. Therefore, our findings suggest that although listeners can exhibit tonal hierarchies of unfamiliar musical culture based on the distribution of tones^53, 55–57^, such a tonal hierarchy does not indicate sensitivity to tonal system information, and thus cannot facilitate tonal structural cognition in listeners.

In conclusion, the present study revealed reduced sensitivity to musical emotion in amusia. However, this finding does not indicate that amusics lack the ability to respond to musical emotion entirely, because emotional connotations of music can be conveyed by both tonal system information and acoustic or psychoacoustic cues. Thus, despite their reduced sensitivity to emotion conveyed by tonal cues, amusics should still be able to use psychoacoustic cues such as tempo, intensity, pitch height, and timbre to judge emotion in daily music listening. Given the universality of using psychoacoustic cues to respond to musical emotion, future studies are needed to further explore the relative contribution of psychoacoustic and tonal system cues to emotional judgment. These investigations may shed further light on the issue concerning whether and to what extent that sensitivity to musical emotion is universal across different populations.

## Methods

### Participants

Fifty-two nonmusicians (26 amusics and 26 controls) were recruited by means of an advertisement posted on the bulletin board system of universities in Shanghai and Shenzhen, China. All participants were native speakers of Mandarin with Han Chinese ethnicity so as to control for the effects of musical enculturation and exposure to Western tonal music. None of the participants had received extracurricular music training, and none reported to have previous exposure to or familiarity with Indian music or instruments. Participants were diagnosed as amusic if scored 65 or below on the three pitch-based subtests of the MBEA, i.e., scale, contour, and interval subtests^6^, and below 78% correct on the MBEA global score, which represents 2 standard deviations below the mean score of normal controls^42^. The two groups were matched in age, years of education, and sex, but differed in the scores of the MBEA (see Supplementary Table 1). The MBEA scores were expressed as the number of correct responses out of 30. As can be seen, the average scores of the amusic group significantly differed from the control group in the melodic subtests (amusics: mean = 18, SD = 1.85, range: 13–20; controls: mean = 28, SD = 1.35, range: 26–30) and the global score of the MBEA (amusics: mean = 19, SD = 1.30, range: 16–21; controls: mean = 28, SD = 1.17, range: 25–30). All participants were right-handed, with normal hearing, and no history of psychiatric or neurological diseases. Ethical approval was obtained from Shanghai Normal University, Northwestern University, and the Chinese University of Hong Kong, and all methods were performed according to the relevant guidelines and regulations. All subjects gave written informed consent prior to participating.

### Stimuli and procedure

Sixteen novel monophonic melodies (8 composed in Western music and 8 in Indian music systems, ranging from 10 to 15 s) selected from Wong *et al*.^40^ were used for this experiment. The melodies were played on both piano and sitar. Therefore, four experimental conditions were included in the present study: Western-piano, Western-sitar, Indian-piano, and Indian-sitar conditions. In order to create overall greater tension response, we included three types of endings (tonic, in-key, or out-of-key) for each melody, which constituted a hierarchy of stability of the tones^58^. It is worth noting that the second, fourth, sixth, or seventh scale tone was used as the in-key ending tones for Western melodies, whereas the second, third, fourth, sixth, or seventh scale tone was used as the in-key ending tones for Indian melodies. As a result, there were a total of 160 melodies (80 for Western melodies, and 80 for Indian melodies) in the present study.

The Western and Indian melodies were matched for tempo, meter, and tonic of key^40^. The only difference was that Western melodies were written in the major and minor scales, while Indian melodies were written in the Bhairav and Todi scales. Because Indian scales are not fixed to particular frequencies, their scale names can be compared to a moveable *do* system of Western music. For instance, taking C as the tonic, the C major scale contains C, D, E, F, G, A, and B, which are named as Do, Re, Mi, Fa, Sol, La, and Ti, while the Indian Bhairav scale is C, D^b^, E, F, G, A^b^, and B, which are named as Sa, Komal Re, Ga, Ma, Pa, Komal Dha, and Ni. Likewise, the C harmonic minor scale contains C, D, E^b^, F, G, A^b^, and B, while the Indian Todi scale includes C, D^b^, E^b^, F^#^, G, A^b^, and B. Thus, there is an overlap of scale tones in Western and Indian music. Our design ensured that C and D tones were used as the tonic for both Bhairav and Todi scales, given that our Western melodies were written in C and D major or minor keys.

We used the MIR toolbox 1.6.1 to extract parameters related to timbre and tonality for each melody. Although the MIR toolbox is specific to Western tonal music in the extraction of tonality parameters, we still extracted tonality parameters for Indian music in order to compare the strategies the two groups used in rating tension, given the overlap of the used tones between Western and Indian scales and the cognitively based perceptual bias towards familiar musical culture^59^. For each melody, 14 parameters related to timbre and 4 parameters related to tonality were extracted (see Supplementary Stimuli). For example, roughness, related to sensory dissonance, corresponds to the “beating” phenomenon^60^, and previous studies have reported that amusics have normal sensitivity to this acoustic cue^16^; spectral flux refers to a measure of the fluctuation of the spectrum over time^61, 62^; irregularity of a spectrum indicates the degree of variation of the successive peaks of the spectrum^63^; zero-crossing rate is a measure of the number of times that the amplitude of the signals crosses the value of zero^64^; chromagram refers to the distribution of the energy along the frequencies, and centroid of the chromagram is an estimate of the fundamental frequency^48^; HCDF is the flux of the tonal centroid, and it can detect not only harmonic recognition, but also other changes in the harmonic content such as a strong melody^65, 66^. We calculated the mean, standard deviation of the timbre and tonality parameters for the four experimental conditions (see Supplementary Table 2). Two-sample *t*-tests were conducted to compare these parameters between the piano and sitar conditions in Western and Indian music. For timbre, both Western and Indian melodies exhibited significant differences between piano and sitar conditions in all parameters (*p*s < 0.05) but attack time (*p* = 0.10 for Western melodies, and *p* = 0.11 for Indian melodies). This may be due to the complexity of attack time, as it reflects not only features of timbre, but also temporal characteristics and intensity. For tonality in Western melodies, piano and sitar conditions did not differ in key clarity (*p* = 0.19) or key strength (*p* = 0.90), but differed in chromagram centroid and HCDF (all *p*s < 0.0001). These differences may be attributed to the chroma features, which are usually called chromagram^67^, a well-established tool for analyzing and comparing harmonic-based Western music^68^. Although the chroma features are relatively invariant to changes in timbre, they are affected by changes in instrumentation^68^, thus influencing the accuracy of HCDF^67^.

The Western and Indian melodies were presented in two blocks. The trials in each block were presented in a pseudo-randomized order with the constraints that 1) a given melody with different endings was separated by more than six trials, and 2) a given ending was separated by more than three trials. During the experiment, participants listened to the melodies and rated the tension evoked by these melodies. Following our previous study^40^, participants were asked to provide a tension response at the end of each melody. Since participants had no prior music training, they were given a short definition of musical tension as states of conflict, instability, dissonance, or uncertainty created by musical events^69^. For explanation, we used viewers’ expectation for plots in a movie as an analogy. When watching a movie, one often builds up expectations from one plot to the next. Suspension and resolution of the plots in a movie would create changes in tension, which may be considered as states of conflict or instability. Four practice trials were given to the participants to familiarize them with the procedure and stimuli. The whole experiment lasted less than an hour.

## Electronic supplementary material

Dataset 1
