# Supplementary material for: Sensitivity to musical emotion is influenced by tonal structure in congenital amusia
Source: Sci Rep. 2017 Aug 8;7:7624. doi: 10.1038/s41598-017-08005-x (PMC5548738; doi:10.1038/s41598-017-08005-x)
Supplement: Supplementary file 1 — Dataset 1 [file 41598_2017_8005_MOESM1_ESM.doc]

**Sensitivity to musical emotion** **is influenced by tonal structure in congenital amusia**

Cunmei Jiang^1,2 *^, Fang Liu^3^, Patrick C. M. Wong ^4,5*^

^1^ Music College, Shanghai Normal University, Shanghai, China

^2^ Institute of Psychology, Shanghai Normal University, Shanghai, China

^3^ School of Psychology and Clinical Language Sciences, University of Reading, Reading, UK

^4^ Department of Linguistics and Modern Languages and Brain and Mind Institute, The Chinese University of Hong Kong, Hong Kong SAR, China

^5^ The Chinese University of Hong Kong – Utrecth University Joint Center for Language, Mind and Brain

Correspondence to:

Dr. Cunmei Jiang

Music College

Shanghai Normal University

100 E. Guilin Road

Shanghai, 200234, China

Tel: 0086-21-64322990; Fax: 0086-21-64322935

Electronic mail: [cunmeijiang@126.com](mailto:cunmeijiang@126.com)

Or

Patrick C. M. Wong,

Department of Linguistics and Modern Languages,

The Chinese University of Hong Kong,

Room G03, Leung Kau Kui Building,

Shatin, N.T., Hong Kong

E-mail: p.wong@cuhk.edu.hk

# Supplementary Stimuli

Using the MIR toolbox 1.6.1 (Lartillot, Toiviainen, & Eerola, 2008), we extracted 14 timbre-related parameters and 4 tonality-related parameters for each melody. The 14 timbre-related parameters are as follows:

1. **Spectral centroid** is the balancing point of a spectrum, calculated as the weighted mean of the frequencies present in the signal. Its curve is similar to the brightness curve, giving rise to a sensation of sound "brightness"(Grey & Gordon, 1978).
2. **Spectral spread** measures the concentration of a spectrum around its centroid. A “small” spread value corresponds to a spectrum highly concentrated near its centroid, which is associated with a "bright" sound in perception (Burred & Lerch, 2004).
3. **Roughness** corresponds to the "beating" phenomenon (Plomp & Levelt, 1965), measured by adding the beating provoked by every two energy peaks in the spectrum (Terhardt, 1974), which is correlated with dissonance and unpleasantness in perception (Coutinho & Dibben, 2013).
4. **Brightness** indicates the amount of high-frequency content in a sound (Paschotta, 2008). Increased brightness makes a sound more dramatic (sounding happier or angrier) (Wu, Horner, & Lee, 2014).
5. **Skewness** measures the asymmetry of a spectrum’s distribution around its mean value. Sad music is usually associated with high skewness values (Laurier, 2011).
6. **Kurtosis** measures the classical power spectrum density, indicating the peakiness of the frequency distribution (Vrabie, Granjon, & Serviere, 2003). Relaxed music is usually associated with high kurtosis values (Laurier, 2011).
7. **Roll-off** is the frequency that splits the signal energy into two parts using a threshold in energy. Sad music usually has a low value of spectral roll-off (Laurier, 2011).
8. **Spectral entropy** is a measure of disorganization, and can be used to measure spectral flatness of a spectrum. Increased spectral entropy makes music sound more like noise (Toh, Togneri, & Nordholm, 2005).
9. **Spectral flatness** describes how flat a spectrum is, calculated based on the mean of the power spectral density components, and is negatively correlated with noisy category (Laurier, 2011).
10. **Attack time** is the duration from the onset of the signal until it reaches its stable part (Peeters, McAdams, & Herrera, 2000). It is strongly associated with brightness: the shorter the attack time is, the coarser the signal sounds (Wu et al., 2014).
11. **Spectral** **irregularity** measures the jaggedness of a spectrum, and is related to emotions such as scary and shy (Chau, Wu, & Horner, 2014).
12. **Mel-frequency cepstral coefficient (****MFCC)** measures the spectral shape of a sound, and divides the signal into frames (Lartillot et al., 2008). The MFCC does not convey very intuitive meaning per se (Lartillot et al., 2008), but makes sounds discernable, and thus frequently used in speech recognition (Li, Pao, & Kuan, 2013; Tyagi & Wellekens, 2005) and music information retrieval (Sigurdsson, Petersen, & Lehn-Schiøler, 2006; Tzanetakis & Cook, 2002).
13. **Zero-crossing rate** is a measure of the number of times that the amplitude of a signal crosses the value of zero (Bachu, Kopparthi, Adapa, & Barkana, 2008). Noisy sounds have higher zero-crossing rates than periodic sounds (Laurier, 2011).
14. **Spectral flux** refers to a measure of the fluctuation of a spectrum over time (McAdams, Winsberg, Donnadieu, De Soete, & Krimphoff, 1995). A low spectral flux gives rise to relaxed feelings, whereas a high spectral flux (together with other factors such as spectral centroid) is associated with happy feelings (Laurier, 2011).

The following are 4 tonality-related parameters:

- 1. **Chromagram centroid**. Chromagram refers to the distribution of the energy along the frequencies, and the centroid of the chromagram is an estimate of the fundamental frequency (Lartillot et al., 2008). A high value of the chromagram centroid is associated with positive valence (Yang & Chen, 2011).
  2. **Key clarity** estimates the strength of the frame in association with key (Yang & Chen, 2011). The highest strength associated with the best key makes music sound steady and smooth.
  3. **K****ey strength** estimates the tonality of a musical piece by calculating the cross-correlation of its chromagram with the distribution associated with each possible tonality (Krumhansl, 1990; Lartillot, Toiviainen, & Eerola, 2008). The higher the key strength, the more steady and smooth the music feels.
  4. **Harmonic Change Detection Function (HCDF)** is the flux of the tonal centroid (Harte, Sandler, & Gasser, 2006). A low value of the HCDF means that no substantial harmonic differences were detected between consecutive frames, which would decrease abrupt impression (Degani, Dalai, Leonardi, & Migliorati, 2015).

**Supplementary Table 1.** Participants’ characteristics and mean scores of the MBEA in the amusic and control groups. *MBEA* scores are expressed as the number of correct responses out of 30.

|  | **Amusic (*n* = 26)** | **Control (*n* = 26)** | ***t*-test** |
| --- | --- | --- | --- |
| Mean age | 25 (1.90) | 24 (2.08) | *n.s.* |
| Sex | 8M, 18F | 8M, 18F |  |
| Hours voluntary music listening per day | 0.55 (0.42) | 0.51 (0.48) | *n.s.* |
| Years education | 18 (1.48) | 17 (1.85) | *n.s.* |
| Melodic subtests of the MBEA | 18 (1.85) | 28 (1.35) | *p* < .001 |
| Global score of the MBEA | 19 (1.30) | 28 (1.17) | *p* < .001 |

*Note*: F = female; M = male. Standard deviation values are shown in parentheses.

**Supplementary Table 2.** Mean, standard deviation of the timbre and tonality parameters extracted with the MIR Toolbox for the four experimental conditions.

|  |  | **Western-piano** | **Western-sitar** | **Indian-piano** | **Indian-sitar** | | |
| --- | --- | --- | --- | --- | --- | --- | --- |
| **Parameters related to timbre** | Attack time | 0.05 (0.00) | 0.05 (0.00) | 0.05 (0.00) | | 0.05 (0.00) |  |
| **Parameters related to tonality** | Spectral centroid  Brightness  Spectral spread  Spectral skewness  Spectral kurtosis  Roll off  Entropy of spectrum  Spectral flatness  Roughness*  Spectral irregularity  MFCC  Zero crossing rate  Spectral flux  Chromagram centroid  Key clarity  Key strength | 1401 (242.10)  0.18 (0.01)  2550 (484.81)  5.80 (0.59)  44.47 (7.88)  3222 (1182)  0.53 (0.01)  0.07 (0.02)  489.23 (17.53)  0.59 (0.07)  0.20 (0.05)  655.40 (60.02)  245.12 (40.57)  70.54 (0.94)  0.61 (0.01)  0.00 (0.00) | 2667 (145.61)  0.47 (0.03)  3312 (108.58)  2.93 (0.18)  14.03 (1.27)  7014 (365.15)  0.68 (0.02)  0.13 (0.01)  730.33 (97.51)  0.53 (0.07)  -0.09 (0.05)  1401 (136.93)  401.98 (63.96)  73.65 (1.10)  0.60 (0.03)  0.00 (0.00) | 1221 (45.00)  0.19 (0.00)  2076 (64.52)  6.44 (0.20)  54.51 (3.13)  2435 (144.05)  0.54 (0.01)  0.05 (0.00)  504.53 (63.63)  0.73 (0.09)  4.16 (0.21)  676.28 (28.28)  268.20 (28.49) | | 2410 (78.55)  0.43 (0.02)  2960 (75.58)  3.09 (0.13)  15.95 (1.10)  6200 (242.13)  0.68 (0.01)  0.11 (0.01)  765.95 (67.18)  0.62 (0.07)  1.86 (0.15)  1254 (75.68)  446.30 (45.00) |  |
|  | HCDF | 0.06 (0.02) | 0.09 (0.02) |  | |  |  |

***** The abscissae position of the detected peak of spectrum.

**References**

Bachu, R. G., Kopparthi, S., Adapa, B., & Barkana, B. D. (2008). *Separation of voiced and unvoiced using zero crossing rate and energy of the speech signal.* Paper presented at the American Society for Engineering Education (ASEE) Zone Conference Proceedings, Pittsburgh, Pennsylvania.

Burred, J. J., & Lerch, A. (2004). Hierarchical Automatic Audio Signal Classification. *Journal of the Audio Engineering Society 52*, 724-739.

Chau, C. J., Wu, B., & Horner, A. (2014). *Timbre Features and Music Emotion in Plucked String, Mallet Percussion, and Keyboard Tones.* Paper presented at the In Proceedings of the 2014 International Computer Music Conference, Athens, Greece.

Coutinho, E., & Dibben, N. (2013). Psychoacoustic cues to emotion in speech prosody and music. *Cognition & emotion, 27*(4), 658-684.

Degani, A., Dalai, M., Leonardi, R., & Migliorati, P. (2015). *Harmonic Change Detection for musical chords segmentation.* Paper presented at the In Multimedia and Expo (ICME), 2015 IEEE International Conference, Torino, Italy.

Grey, J. M., & Gordon, J. W. (1978). Perceptual effects of spectral modifications on musical timbres. *Journal of the Acoustical Society of America, 63*(5), 1493-1500.

Harte, C., Sandler, M., & Gasser, M. (2006). *Detecting harmonic change in musical audio.* Paper presented at the Proceedings of the 1st ACM workshop on Audio and music computing multimedia, Santa Barbara, California, USA.

Krumhansl, C. L. (1990). *Cognitive foundations of musical pitch*. New York, NY: Oxford University Press.

Lartillot, O., Toiviainen, P., & Eerola, T. (2008). A matlab toolbox for music information retrieval. In C. Preisach, Burkhardt, H., Schmidt-Thieme, L., Decker, R. (Ed.), *Data analysis, machine learning and applications* (pp. 261-268). Berlin, Germany: Springer.

Laurier, C. (2011). *Automatic Classification of Musical Mood by Content-Based Analysis.* Ph.D. thesis Universitat Pompeu Fabra, Barcelona.

Li, Y. J., Pao, T. L., & Kuan, C. M. (2013). *Emotional corpus based on MFCC and the coefficient of correlation.* Paper presented at the In Orange Technologies (ICOT), 2013 International Conference Tianwan.

McAdams, S., Winsberg, S., Donnadieu, S., De Soete, G., & Krimphoff, J. (1995). Perceptual scaling of synthesized musical timbres: Common dimensions, specificities, and latent subject classes. *Psychological research, 58*(3), 177-192.

Paschotta, R. (2008). spectral brightness *in the Encyclopedia of Laser Physics and Technology* (1st ed.): Wiley-VCH.

Peeters, G., McAdams, S., & Herrera, P. (2000). *Instrument sound description in the context of mpeg-7.* Paper presented at the Proceedings of the 2000 International Computer Music Conference, Citeseer.

Plomp, R., & Levelt, W. J. (1965). Tonal consonance and critical bandwidth. *The journal of the Acoustical Society of America, 38*(4), 548-560.

Sigurdsson, S., Petersen, K. B., & Lehn-Schiøler, T. (2006). *Mel frequency cepstral coefficients: An evaluation of robustness of mp3 encoded music.* Paper presented at the In Seventh International Conference on Music Information Retrieval (ISMIR), Victoria, Canada.

Terhardt, E. (1974). On the Perception of Periodic Sound Fluctuations (Roughness). *Acustica, 30*(4), 201-213.

Toh, A. M., Togneri, R., & Nordholm, S. (2005). *Spectral entropy as speech features for speech recognition.* Paper presented at the Proceedings of PEECS.

Tyagi, V., & Wellekens, C. (2005). *On desensitizing the Mel-Cepstrum to spurious spectral components for Robust Speech Recognition.* Paper presented at the In Acoustics, Speech, and Signal Processing Proceedings (IEEE International Conference 2005), Hong Kong.

Tzanetakis, G., & Cook, P. (2002). *Musical genre classification of audio signals.* Paper presented at the IEEE Transactions on speech and audio processing.

Vrabie, V., Granjon, P., & Serviere, C. (2003). *Spectral kurtosis: from definition to application.* Paper presented at the 6th IEEE International Workshop on Nonlinear Signal and Image Processing, Grado-Trieste, Italy.

Wu, B., Horner, A., & Lee, C. (2014). *Musical timbre and emotion: The identification of salient timbral features in sustained musical instrument tones equalized in attack time and spectral centroid.* Paper presented at the In Proceedings of the 2014 International Computer Music Conference, Athens, Greece.

Yang, Y. H., & Chen, H. H. (2011). *Music emotion recognition*: CRC Press.
